# Hypoxic Induction of the Regulator of G-Protein Signalling 4 Gene Is Mediated by the Hypoxia-Inducible Factor Pathway

**DOI:** 10.1371/journal.pone.0044564

**Published:** 2012-09-07

**Authors:** Sam W. Z. Olechnowicz, Anthony O. Fedele, Daniel J. Peet

**Affiliations:** 1 School of Molecular and Biomedical Science, University of Adelaide, Adelaide, Australia; 2 ARC Special Research Centre for the Molecular Genetics of Development, University of Adelaide, Adelaide, Australia; 3 Lysosomal Diseases Research Unit (LDRU), SA Pathology, North Adelaide, Australia; University of Nebraska Medical Center, United States of America

## Abstract

The transcriptional response to hypoxia is largely dependent on the Hypoxia Inducible Factors (HIF-1 and HIF-2) in mammalian cells. Many target genes have been characterised for these heterodimeric transcription factors, yet there is evidence that the full range of HIF-regulated genes has not yet been described. We constructed a TetON overexpression system in the rat pheochromocytoma PC-12 cell line to search for novel HIF and hypoxia responsive genes. The *Rgs4* gene encodes the Regulator of G-Protein Signalling 4 (RGS4) protein, an inhibitor of signalling from G-protein coupled receptors, and dysregulation of *Rgs4* is linked to disease states such as schizophrenia and cardiomyopathy. *Rgs4* was found to be responsive to HIF-2α overexpression, hypoxic treatment, and hypoxia mimetic drugs in PC-12 cells. Similar responses were observed in human neuroblastoma cell lines SK-N-SH and SK-N-BE(2)C, but not in endothelial cells, where *Rgs4* transcript is readily detected but does not respond to hypoxia. Furthermore, this regulation was found to be dependent on transcription, and occurs in a manner consistent with direct HIF transactivation of *Rgs4* transcription. However, no HIF binding site was detectable within 32 kb of the human *Rgs4* gene locus, leading to the possibility of regulation by long-distance genomic interactions. Further research into *Rgs4* regulation by hypoxia and HIF may result in better understanding of disease states such as schizophrenia, and also shed light on the other roles of HIF yet to be discovered.

## Introduction

Oxygen is critical for the survival of metazoan cells, and as such there is an adaptive response to chronic cellular hypoxia, involving activation of the Hypoxia Inducible Factor (HIF) pathway. HIF is a heterodimeric transcription factor which binds at over 100 genomic sites to activate transcription of hypoxia-adaptive genes [1], [2]. HIF is comprised of two basic-Helix-Loop-Helix/Per-Arnt-Sim domain (bHLH/PAS) proteins, known as HIF-α and HIF-β [3], which dimerise in the nucleus to activate transcription [4]. HIF-α subunits are hypoxia responsive, while HIF-β (alternatively known as ARNT) is constitutively expressed and unresponsive to hypoxia.

There are three genes encoding HIF-α subunits: *Hif1a*, which encodes HIF-1α protein [5]; *Epas1*, which encodes HIF-2α protein, also known as HIF-Like Factor (HLF) or Endothelial Per-ARNT-Sim protein-1 (EPAS1) [6]–[8]; and *Hif3a*, which encodes various splice variants of HIF-3α protein [9], [10]. However, only HIF-1 and HIF-2 are active transcription factors, formed by respective dimerisation between HIF-1α or HIF-2α with HIF-β. HIF-1α and HIF-2α are not redundant, and have differing physiological roles, as established by several independent transgenic and knockout mouse lines [11]–[15]. In oxygenated cells, HIF-α subunits are rapidly degraded and inactivated by the oxygen-dependent PHD and FIH hydroxylases [16]–[18], but in hypoxic conditions they are stabilised and transactivate specific genes such as *Vegf* [19] and *Dec1* [20]. Both forms of HIF bind directly to a DNA sequence known as the Hypoxia Response Element (HRE), with a consensus sequence of RCGTG [1], [3], [21]. Known HREs are most commonly found within 2kb of the target gene transcription start site (TSS) [22], however there are examples of more distant functional HREs, such as that of the *Phd3* gene which is found over 10 kb from its TSS [21]. Differences between HIF-1 and HIF-2 target selection are caused by interaction with other transcription factors or chromatin context, rather than through DNA target sequence selection [23], [24].

*Rgs4* is a member of a large family of genes encoding RGS G-Protein signalling regulators, which bind to GTP-bound G_α_ and increase the rate of GTP hydrolysis, causing reduced signal transduction from the associated G-protein coupled receptors [25]–[27]. *Rgs4* is expressed predominantly in neural and vascular cells [28], [29], and the *Rgs4* locus has been linked to disease states relating to both types of cell, notably in schizophrenia [30]. Although the causes of schizophrenia are not yet understood, one major theory involves dysregulation of dopamine signalling [31], so it is interesting that RGS4 protein is capable of inhibiting signal transduction from the D_2_ dopamine receptor [32], [33]. Notably, schizophrenia risk is also linked to reduced oxygen availability during foetal and post-natal development [34]–[37]. *Rgs4* is related to *Rgs5* in sequence, function and regulation, and both gene products have roles in cardiovascular development and homeostasis [38]. RGS4 and RGS5 proteins have been previously shown to be protected from degradation by hypoxia and nitric oxide [38], [39], while *Rgs4* also appears in a list of hypoxia upregulated genes in the SK-N-BE(2)C cell line [40]. However, no mechanistic link between *Rgs4* gene regulation and hypoxia has been reported.

Although there are numerous characterised HIF target genes [1], a majority of these have been discovered in a limited number of specific cell types, particularly breast and kidney cancer cell lines. The PC-12 rat pheochromocytoma, SK-N-SH and SK-N-BE(2)C human neuroblastoma cell lines all stabilise and activate both HIF-1α and HIF-2α protein endogenously in hypoxia [41], [42], and are derived from tumours of the sympathetic nervous system. Therefore, to identify novel HIF-regulated target genes we developed an inducible expression system to selectively express either HIF-1α or HIF-2α in PC-12 rat pheochromocytoma cells, and performed siRNA-targeted knockdown of endogenous HIF-1α or HIF-2α in neuroblastoma cells. We report gene expression analysis demonstrating that *Rgs4* is a cell-type specific hypoxia inducible target in PC-12 and neuroblastoma cell lines, under the control of HIF-1 and HIF-2. The use of hypoxia mimetics targeting the HIF hydroxylases and siRNA knockdown experiments confirm a role for the endogenous HIFs in hypoxic regulation of *Rgs4*, but not *Rgs5*.

## Methods

### Tissue Culture

PC-12 rat pheochromocytoma cells [43] were grown in Dulbecco’s Modified Eagle Medium (DMEM) supplemented with 10% Horse Serum and 5% Fetal Calf Serum (FCS). Neuro-2A mouse neuroblastoma [44] and SK-N-SH human neuroblastoma cells [45] were grown in Minimal Essential Medium (MEM) supplemented with 10% FCS. SK-N-BE(2)C human neuroblastoma cells [46] were grown in RPMI-1640 supplemented with 10% FCS. Human Umbilical Vein Endothelial Cells (HUVECs) were a kind gift from Dr Claudine Bonder (Institute of Medical and Veterinary Science, South Australia) [47]. Cells were maintained at 37°C and 5% CO_2_, and passaged with trypsinisation when near-confluent, and hypoxic conditions (<0.5% O_2_) were achieved by sealing the culture vessel inside an airtight container along with an AnaeroGen sachet (Oxoid). Cells were treated with 1 mM dimethyloxalylglycine (DMOG), 100 µM 2,2′-dipyridyl (DP) or DMSO as a negative (vehicle) control.

### Plasmid Construction

pEF/TetON/IRES/PURO was constructed by transferring the *Eco*RI and *Bam*HI (blunted) fragment from pTET-On (Clontech) into *EcoRI* and *Eco*RV digested pEF/IRES/PURO [48]. To construct pEF/TetON/IRES/NEO, pEF/TetON/IRES/PURO was digested with *Nhe*I and *Not*I and the 1 kb fragment inserted into *Nhe*I and *Not*I digested pEF/IRES/NEO. pTR/DC/EYFP was generated by cloning the *Ssp*I and *Nco*I fragment from pTR5-DC/GFP [49] into *Ssp*I and *Nco*I digested pIRES-EYFP (Clontech). To construct pTR/HIF-2α/DC/EYFP, pHIF-2α/SL301 was digested with *Hpa*I and *Eco*RV and the 3 kb fragment ligated into *Pme*I digested pTR/DC/EYFP. To construct pTR/HIF-1α/DC/EYFP, pET-32a/HIF-1α was digested with *Bgl*II and *Bam*HI and the 2.5 kb fragment cloned into *Bgl*II digested pTR/DC/EYFP. The resultant construct was termed pTR/HIF-1α C-terminus/DC/EYFP. To introduce the N-terminus of HIF-1α, pET-32a/HIF-1α was digested with *Bgl*II and the 0.1 kb fragment transferred into *Bgl*II digested pTR/HIF-1α C-terminus/DC/EYFP. To then construct pTR/HIF-1αN803A/DC/EYFP, pEFBOS/HIF-1αN803A was digested with *Afl*II and *Hpa*I and the 1.3 kb fragment purified and inserted into pTR/HIF-1α/DC/EYFP (from which a 1.3 kb fragment was also released).

### Tet-ON PC-12 Cell Line Development

The parental monoclonal PC-12/TetON/NEO cell line was established from PC-12 cells transfected with 10 µg of pEF/TetON/IRES/NEO and supplementation of the medium with 200 µg/mL of G418. The individual colony displaying the greatest reverse tetracycline transactivator (rtTA) activity was maintained for the establishment of subsequent lines. PC-12/TetON/NEO cells were transfected with 5 µg of pTR/DC/EYFP, pTR/HIF-2α/DC/EYFP or pTR/HIF-1αN803A/DC/EYFP. After multiple passaging and exposure to 2 µg/mL doxycycline for 16 hours, FACS was performed and all fluorescing cells were collected. After cultivation, these cells were once again exposed to 2 µg/mL of doxycycline for 16 hours, FACS performed and the highest fluorescing cells selected (approximately 5%). Monoclonal lines were generated by limiting dilution, and then HRE activity was analysed using reporter assays and HIF-1α and HIF-2α protein was detected via immunoblotting. Separation of proteins for immunoblotting was performed via the electrophoresis of equivalent amounts of whole cell extracts through 7.5% acrylamide tris/glycine SDS PAGE gels. The membrane was blocked for 1 hour in 10% milk, phosphate-buffered saline, and 0.1% Tween 20 (PBST), incubated overnight with 1/500 dilution of primary antibody (polyclonal rabbit anti HIF-1α amino acids 727–826 or polyclonal rabbit anti HIF-2α amino acids 612–823), diluted in 10% milk, phosphate-buffered saline, and 0.1% Tween 20. The membrane was washed three times for 10 minutes in phosphate-buffered saline and 0.1% Tween 20 prior to incubation in 2.5% milk, PBST and 1/2000 (DAKO) or 1/20000 (Pierce) dilution of horse radish peroxidase conjugated goat anti rabbit immunoglobulin for 1 hour. The membrane was washed three times for 10 minutes in PBST and blotted dry and analysed by chemiluminescence with autoradiography.

### Microarray

Four 175 cm^2^ flasks of PC-12/TetON/EYFP and PC-12/TetON/HIF-2α/EYFP cells were exposed to 2 mg/mL doxycycline or left untreated for 16 hours for each treatment. Total RNA was prepared, then cDNA synthesis and labelling reactions performed using a mixture of random hexamers, labelled poly dT oligonucleotides and Cy3 and Cy5 dyes, according to the established protocols at the Adelaide Microarray Facility, which have been described previously [50]. Hybridisation of the labelled RNA to 10 K rat oligonucleotide microarray slides (Clive and Vera Ramaciotti Centre for Gene Function Analysis, Australia) was performed according to the manufacturer’s instructions. Statistical analysis was achieved using the using the Spot v3 plugin (CSIRO, Australia) within the R statistical software package (http://www.R-project.org), as detailed previously [50].

**Figure 1:**
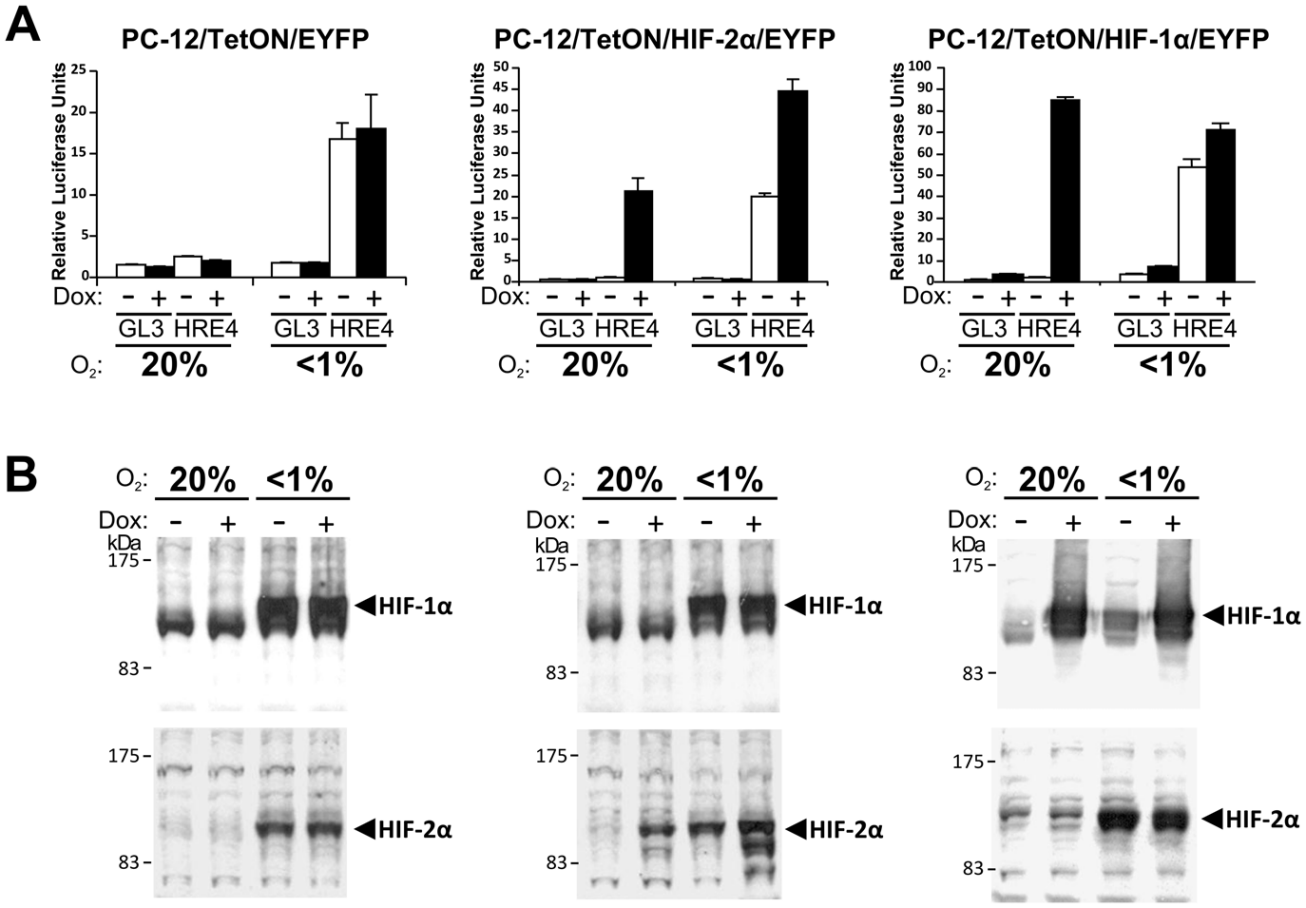
Generation and verification of PC-12 TetON HIF-α inducible expression cell lines. (A) PC-12/TetON/EYFP, PC-12/TetON/HIF-2α/EYFP and PC-12/TetON/HIF-1α/EYFP cells were transfected with a firefly luciferase reporter gene containing 4 copies of the HRE (pHRE4) or a control construct which lacks the HRE (pGL3). 6 hours post transfection, cells were exposed to 20% O_2_ or <1% O_2_ in the presence or absence of 2 µg/mL doxycycline (Dox). After 16 hours, cells were assayed for firefly luciferase activity against a cotransfected internal renilla luciferase control. Results representative of 3 independent experiments performed in triplicate. Error bars represent standard deviation. (B) Cell extracts were prepared from cells treated as in (A), and 20 µg of total protein from each was analysed by immunoblotting with antibodies to HIF-1α (upper immunoblot) or HIF-2α (lower immunoblot).

### Gene Expression Analysis

After the indicated treatment, cells were lysed and RNA extracted with TriReagent (Sigma), as per the manufacturer’s protocol. RNA integrity was assessed by spectrophotometry (Eppendorf BioPhotometer) and gel electrophoresis. For northern blotting, 20 µg of total RNA was run on a formamide/MOPS/1% agarose gel, and transferred to Nytran membrane (Schleicher and Schuell) by passive blotting. ^32^P-dATP labelled DNA probes were generated from cDNA clones, originally made from cDNA fragments amplified by RT-PCR using the following primer sequences: *Rgs4*: ATG TGC AAA GGA CTC GCT GGT, TTA GGC ACA CTG AGG GAC TAG; *Vegf*: GCC TTG CTG CTC TAC CTC CAC, CAA ATG CTT TCT CCG CTC TGA; *β-Actin*: CTG GCA CCA CAC CTT CTA C, GGG CAC AGT GTG GGT GAC.

^32^P-dATP labelled cDNA probes were hybridised to the Nytran membrane overnight at 42°C in 50% formamide with 5x SSC buffer, 50 mM NaPO_4_ (pH 6.5), 5x Denhardt’s solution, 0.1% SDS and 0.3 mg/mL sheared salmon sperm DNA. The membranes were washed twice with 2x SSPE/0.1% SDS at 42°C and once with 0.1x SSPE/0.1% SDS at 65°C, then exposed to a phosphorimaging plate for 48–72 hours before scanning and analysis with a Typhoon Trio instrument (Amersham Biosciences) and QuantityOne software (Bio-Rad). Probes were stripped from membranes by boiling in 10 mM Tris (pH 7.5) and 0.1% SDS for 5 minutes before reprobing.

**Figure 2:**
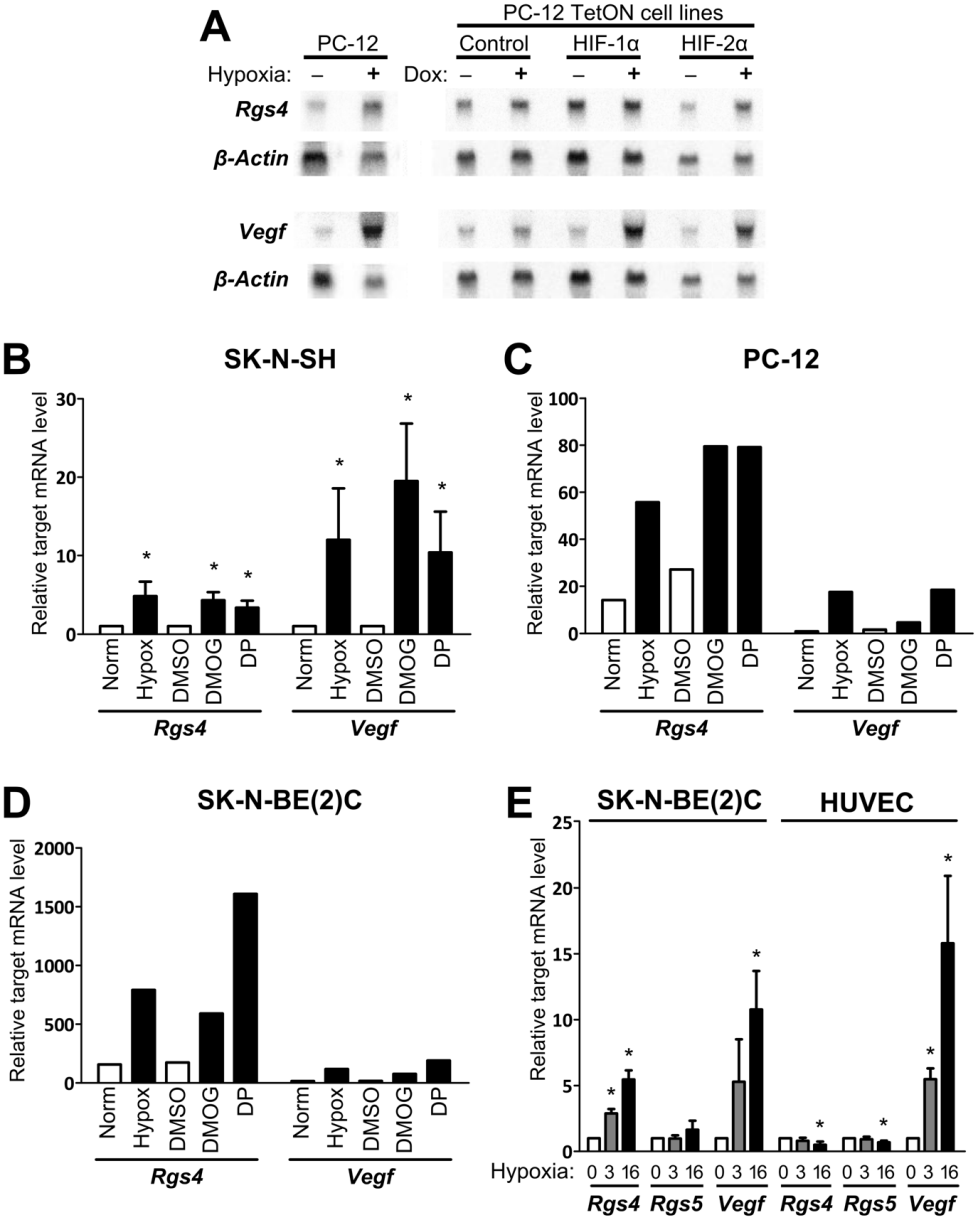
*Rgs4* is responsive to hypoxia and hypoxia-mimetic chemicals. (A) Northern blot of total RNA from parental PC-12 cells with or without 16 hours of hypoxic treatment, and the TetON-Control, TetON-HIF1α and TetON-HIF-2α PC-12 cell lines with or without 16 hours of doxycycline treatment (Dox). Blot shown is representative of either three (*Rgs4* with doxycycline treatments), two (*Rgs4* with hypoxia, *Vegf* with doxycycline) or one (*Vegf* with hypoxia) independent experiments. (B) SK-N-SH cells were treated with normoxia, hypoxia, 0.1% DMSO (vehicle), 1 mM DMOG or 100 µM DP for 16 hours before target gene analysis by qRT-PCR. Mean fold change with standard deviation for three independent experiments is shown. (C) PC-12 and (D) SK-N-BE(2)C cell lines were tested in two independent experiments as in 1(B). Representative experiments (from n = 3) are shown. (E) SK-N-BE(2)C and HUVEC cell cultures were treated with 0 (normoxia), 3 and 16 hours of hypoxia before quantification of *Rgs4*, *Rgs5* and *Vegf* mRNA levels relative to *Polr2a* using qRT-PCR, normalised to normoxic levels. Mean fold change with standard deviation for three independent experiments is shown. Asterisks indicate that the normoxic control lies outside of the 99% confidence interval of relative mean target gene expression.

For quantitative reverse transcription PCR (qRT-PCR), 2 µg of total RNA was used as a template for cDNA synthesis with both random hexamer and poly-dT priming. Quantitative PCR was run in triplicate for each cDNA sample, using Fast SYBR Green Mastermix (Applied Biosystems) as per the manufacturer’s protocol, primers as specified in supplementary material, and the StepOne Plus thermal cycler (Applied Biosystems). Negative controls without reverse transcriptase were run for each experiment. qPCR efficiency was calculated for each primer set to be 0.9<*E*<1.1, and amplification specificity was ensured by gel electrophoresis of PCR products, and melting curve analysis. Target gene expression was calculated relative to reference gene (*Polr2a*) expression for each experiment using the ΔΔCt method and Q-Gene software [51]. For statistical analysis, each independent experiment was normalised to the control to yield fold change in target gene expression, then mean and standard deviation across independent experiments calculated.

For statistical analysis of gene expression data using pooled data from independent biological replicates, the 99% confidence interval was calculated for each treatment, using the mean fold change, standard deviation and number of biological replicates. Statistical significance was assigned to treatment samples for which the control treatment value (set at 1) lies outside of the 99% confidence interval.

**Figure 3:**
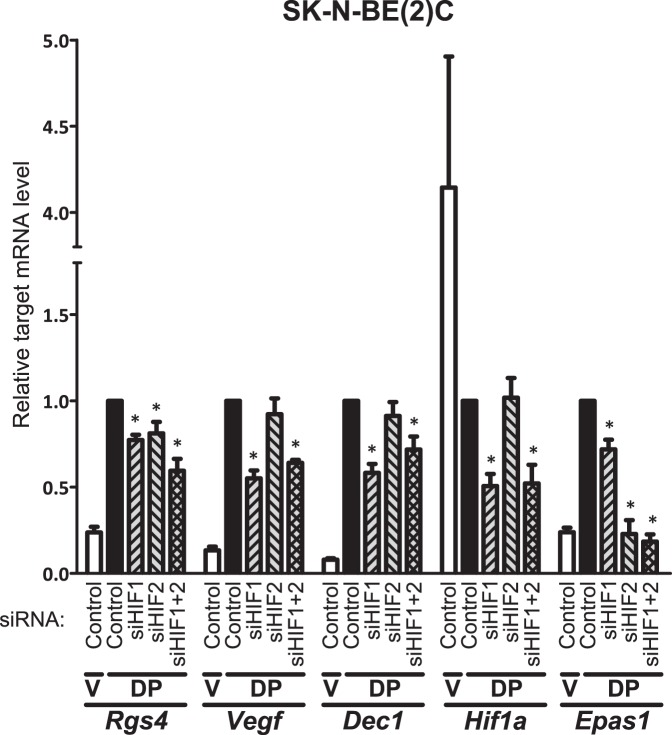
Knockdown of HIF-α subunits impairs response of *Rgs4*. SK-N-BE(2)C cells were transfected twice with 50 nM of negative control siRNA, or siRNA targeted towards *Hif1a* or *Epas1* mRNA (siHIF1_1541 or siHIF2_1599, respectively), or 50 nM each of both targeted siRNAs together. Cells were then grown for 8 hours with 0.1% DMSO vehicle (V) or 100 µM DP before collection and gene expression analysis for *Rgs4*, *Vegf*, *Dec1*, *Hif1a* and *Epas1* expression levels relative to *Polr2a* by qRT-PCR. Mean fold change with standard deviation between three independent experiments is shown, each normalised to the DP-treated level of expression of each gene after negative control siRNA transfection, with asterisks indicating that the DP-treated control lies outside of the 99% confidence interval of relative target gene expression.

### siRNA Knockdown

siRNA duplexes (Qiagen) targeted specific sequences described in [52]. Subconfluent cells were transfected twice sequentially with 50 nM siRNA using siLentFect reagent (Bio-Rad), before replacement of media, and treatment with DP or vehicle as described above. Whole cell extracts from duplicate samples were obtained through lysis of cells in 1x Laemmli buffer, separated by 7.5% SDS PAGE, and transferred to nitrocellulose, and analysed as described above.

**Figure 4:**
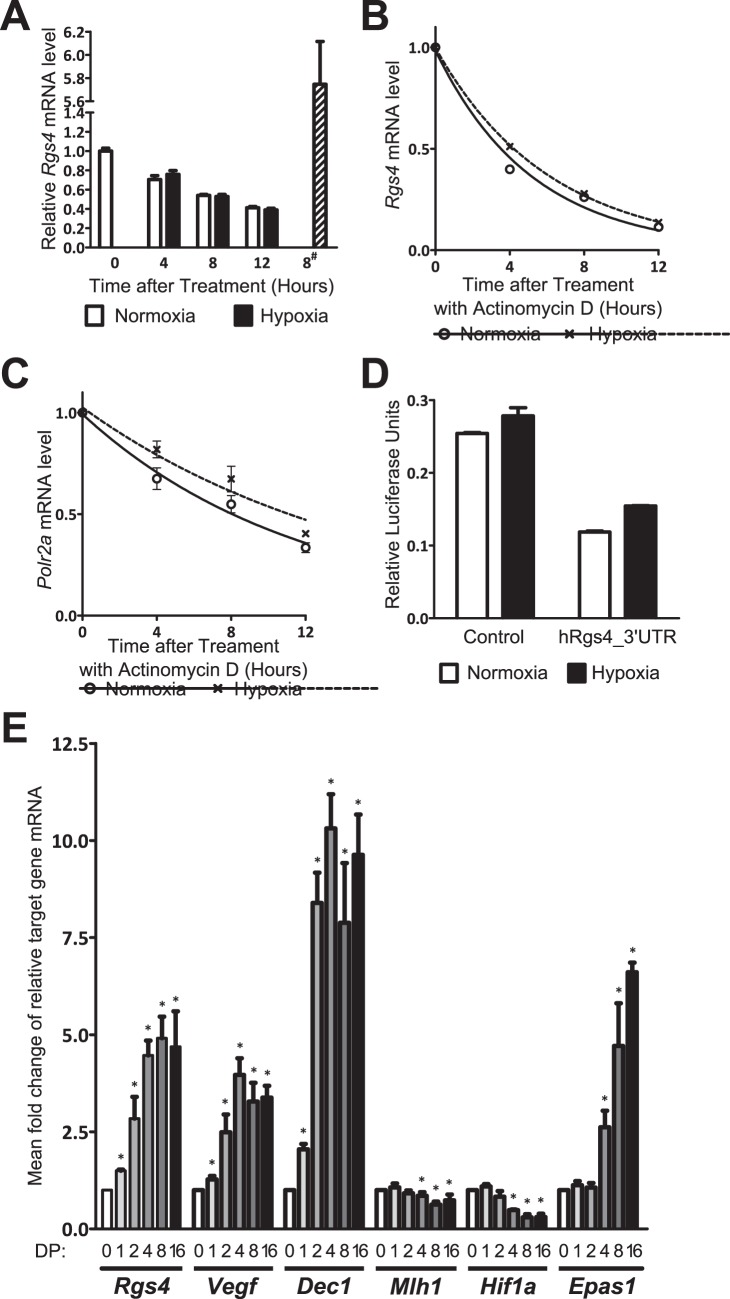
*Rgs4* response is not due to mRNA stability or indirect transcriptional activation. (A) SK-N-BE(2)C cells were treated with 4 µg/ml actinomycin D prior to treatment with 4, 8 or 12 hours of normoxia or hypoxia. Untreated cells were also used to give control normoxic (0 hours) and hypoxic (8^#^ hours) *Rgs4* levels. *Rgs4* mRNA levels were quantified using qRT-PCR relative to *Polr2a*, then normalised to normoxic levels. One representative of *n* = 2 is shown. *Rgs4* (B) and *Polr2a* (C) mRNA levels relative to total RNA were also calculated from C(t) values without normalisation to reference gene relative to normoxia. The mean detection with standard error (*n* = 2) is plotted to give a one-phase decay using GraphPad Prism 5. Estimated mRNA half-lives are described in the text. (D) SK-N-BE(2)C cells were transfected twice with 100 ng of pCI_FL (control) or pCI_FL incorporating the 3′UTR of human *Rgs4* (hRgs4_3′UTR). Mean relative luciferase units with standard deviation from 3 replicate wells of one experiment are shown as a representative of *n* = 3. (E) SK-N-BE(2)C cells were treated with 100 µM DP for 1, 2, 4 8 or 16 hours in comparison to an untreated (0 hours) control. Message levels were quantified by qRT-PCR for indicated transcripts relative to reference gene *Polr2a*, then normalised to normoxic levels. Data is presented as the mean and standard deviation from three independent experiments. Asterisks indicate that control level lies outside of the 99% confidence interval of the DP-treated target gene expression.

### Actinomycin D Treatment

Cells were treated with 4 µg/mL actinomycin D [53], or equivalent DMSO as a negative vehicle control, then incubated in normoxia or hypoxia, and gene expression was analysed by qRT-PCR as described previously. *Rgs4* mRNA level decay was calculated in relation to total RNA levels, as well as after normalisation to reference gene *Polr2a*. Values from two independent experiments without normalisation were entered into Prism (GraphPad) and graphed as an *x*,*y* scatter plot with one phase decay line fitted, constraining the plateau to 0. Calculated half-life ranges at 99% confidence were then computed by Prism software.

**Figure 5:**
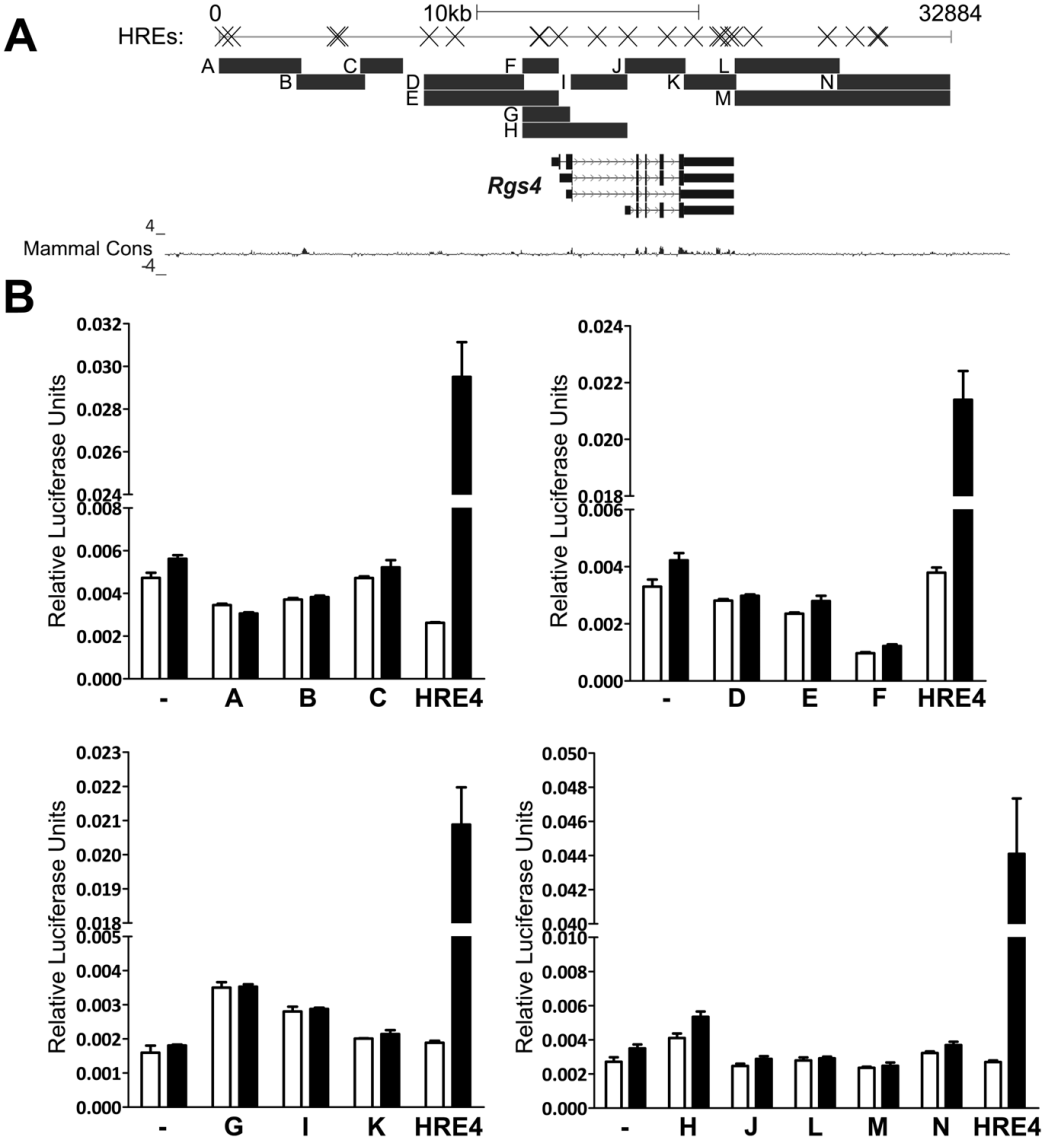
Functional hypoxia responsive elements cannot be detected within 33 **kb of the human** ***Rgs4*** **gene.** (A) Diagram of the human *Rgs4* locus, with HRE-like sequences depicted by crosses, and the regions (A-N) that were cloned by genomic PCR and inserted into pGL3 upstream of the *luc* ^+^ coding sequence. Primer sequences for PCR are listed in Table S1. *hRgs4* splice variants and regions of high conservation within mammals as shown as depicted by UCSC Genome Browser. (B) SK-N-BE(2)C cells were transfected twice with 100 ng of pGL3promoter (-) or pGL3-based reporter plasmids incorporating the genomic sequences depicted in (A), while 25 ng pHRE4 was transfected separately as a positive control for hypoxia treatment. Cells were treated with hypoxia for 16 hours, before relative luciferase expression analysis. Mean relative luciferase units of triplicate wells with standard deviation are depicted, as a representative of three independent experiments.

### Luciferase Reporter Assays

TetON PC-12 cells were transiently transfected using Lipofectamine2000 (Invitrogen) with 100 ng of pRLTK (Promega) and 100 ng of pHRE4 (pHRE_4_GL3, provided by Yoshiaki Fujii-Kuriyama, Tohoku University, Japan [7]) or pGL3 (Promega) and were then left untreated or treated with 2 µg/mL doxycycline, both with or without additional exposure to hypoxia, for 16 hours. For later experiments, SK-N-BE(2)C cells in 24-well trays were transfected twice sequentially over two days with 100 ng of pGL3 or 25 ng of pHRE4 firefly luciferase reporter and 25 ng of phRLCMV renilla luciferase reporter in 0.5 mL serum-free media per well. Sequences flanking the human *Rgs4* gene were cloned by genomic PCR (primer sequences available in supplementary data) and subcloned to the MCS of pGL3. For 3′UTR analysis, *Rgs4* sequence K was subcloned between the *luc* ^+^ coding sequence and polyadenylation signal site and transfected as per the pGL3-based vectors. Cells were then treated with hypoxia or normoxia for 16 hours, and luciferase expression was measured using the Dual Luciferase System (Promega) and luminometer (Promega) as per the manufacturer’s protocol.

## Results

### Generation of TetON Inducible PC-12 Cell Lines and Analysis of Gene Expression by Microarray

In order to investigate novel, cell-type specific HIF target genes, HIF-1α and HIF-2α tetracycline inducible (TetON) monoclonal PC-12 cell lines were established. To characterise HIF induction, cells were transfected with pHRE4 or pGL3 luciferase reporter genes and treated with 2 µg/mL doxycycline or vehicle control, and exposed to normoxia or hypoxia. Cells were assayed for luciferase activity (Figure 1A) and for HIF-1α or HIF-2α protein levels by immunoblotting (Figure 1B).

As anticipated, hypoxia induced HIF-1α and HIF-2α protein expression and HRE-dependent activity in all cell lines. Importantly, doxycycline treatment induced HRE activity in both HIF-inducible cell lines, by inducing the expression of HIF-2α protein in the PC-12/TetON/HIF-2α/EYFP cell line and HIF-1α protein in the PC-12/TetON/HIF-1α/EYFP cell line.

Due to the low inducibility of the HRE reporter gene by HIF-1α in the stable cell lines (data not shown), the HIF-1α N803A mutant which prevents asparaginyl hydroxylation by FIH was used [18], resulting in similar HRE induction to the HIF-2α line (Figure 1A). This was not surprising as overexpression of wildtype HIF-1α protein, but not HIF-2α, has been reported to require hypoxia for full transcriptional activity [54], and HIF-1α has also been shown to be a better substrate for FIH than HIF-2α [55].

Interestingly, although the overexpressed HIF proteins are likely to avoid degradation due to saturation of the PHDs, over expression and stabilisation of one of the HIFs using doxycycline does not appear to stabilise the other endogenous HIF.

### *Rgs4* Gene Expression Profiling

To identify HIF-2α target genes, a cDNA microarray was performed using RNA extracted from PC-12/TetON/HIF-2α/EYFP cells treated with 2 µg/mL of doxycycline or vehicle control for 16 hours. As expected, the transcript most induced by doxycycline treatment of the PC-12/TetON/HIF-2α/EYFP cell line was HIF-2α (a 15.5 fold increase), whereas transcript levels of HIF-1α remained unchanged, validating the array (data not shown). One of the genes showing the greatest induction with HIF-2α overexpression compared to the control cell line was *Rgs4* (2.1 fold). This was comparable to *Vegf*, a well-characterised target gene of both HIF-1 and HIF-2 [8], [19], [56], [57], whose expression was increased 1.9 fold. Hence *Rgs4* expression was further examined by northern blot for response to 16 hours of doxycycline-induced overexpression in both of the HIF TetON PC-12 cell lines (using mRNA from independent samples to those used in the microarray), and also for response to 16 hours of hypoxic treatment in the parental PC-12 cells (Figure 2A). Overexpression of HIF-1α or HIF-2α increased *Vegf* mRNA relative to loading controls as expected, and the effect of HIF-1α was greater than that of HIF-2α. The basal levels of *Rgs4* were altered by the stable transfection of the doxycycline-regulated vectors compared to unmodified PC-12 cells, therefore untreated controls were included for all modified cell-lines. However, some changes in gene expression due to doxycycline-induced HIF-α overexpression may be masked. *Rgs4* mRNA was consistently elevated in both HIF-2α overexpressing and hypoxic parental PC-12 cells compared to the relevant controls (Figure 2A), but no reproducible regulation of *Rgs4* by overexpressed HIF-1α was detected. This is consistent with *Rgs4* being a HIF-2α target in PC-12 cells.

As *Rgs4* displays cell-specific expression, the hypoxic regulation of *Rgs4* in other cell types was investigated, including human neuroblastoma SK-N-SH and SK-N-BE(2)C cells. Total RNA was extracted after normoxic or hypoxic treatment of each cell type, and analysed by northern blot and quantitative RT-PCR for expression *of Rgs4,* and the well characterised HIF target gene *Vegf* (Figure 2B–D). *Vegf* and *Rgs4* mRNA expression was readily detectable and hypoxically induced, with a similar level of response to that observed in parental PC-12 cells (Figure 2B–D). Mouse neuroblastoma Neuro-2A cells were also tested, with similar results (data not shown). Furthermore, treatment of PC-12, SK-N-SH, SK-N-BE(2)C and Neuro-2A cells with hypoxia mimetics dimethyloxallylglycine (DMOG) or 2,2′-dipyridyl (DP) resulted in increased *Rgs4* levels relative to controls. DMOG and DP are both known to mimic the transcriptional hypoxic response by antagonising the HIF hydroxylases PHD1-3 and FIH, resulting in stable and active HIF heterodimers [16], [17]. However, *Rgs4* mRNA was not detectable in 293T or P19 extracts (data not shown). Together these data demonstrate the cell-specific induction of *Rgs4* by hypoxia in pheochromocytoma and neuroblastoma cells, and are consistent with a role of the HIF pathway in this response.

*Rgs5* encodes another member of the RGS domain protein family with high homology to *Rgs4*, and the two genes are separated by only 65 kb on Chromosome 1 according to the UCSC human genome browser [58]. Previous studies have reported that *Rgs5*, but not *Rgs4*, is responsive to hypoxia in Human Umbilical Vein Endothelial Cells (HUVECs) [59]. Therefore, we compared hypoxic induction of *Rgs4* and *Rgs5* in HUVEC cells to the SK-N-BE(2)C neuroblastoma cells which display strong *Rgs4* induction. 3 or 16 hour hypoxic treatment of SK-N-BE(2)C resulted in significant and reproducible increases in *Rgs4* expression (2.88±0.34 and 5.46±0.70 fold, respectively). However, no significant changes in *Rgs5* expression were observed after either 3 hour (0.98±0.24) or 16 hour (1.64±0.68) hypoxic treatment of cells (Figure 2E). Interestingly, neither *Rgs4* nor *Rgs5* were induced by hypoxic treatment of HUVEC cells, but instead a small yet significant decrease in the expression of both *Rgs4* (0.51±0.24) and *Rgs5* (0.69±0.13) was detected after 16 hours of treatment. In contrast, there was a strong induction of the positive control *Vegf*. These results demonstrate that *Rgs4* and *Rgs5* are not hypoxically inducible in HUVECs under the conditions tested.

### Role of Endogenous HIF-1α and HIF-2α in *Rgs4* Hypoxic Induction

In order to directly implicate the endogenous HIF transcription factors in regulation of *Rgs4*, siRNA duplexes targeted at either *Hif1a* or *Epas1* mRNA at previously defined sites [52] were used. SK-N-BE(2)C cells were transfected with either an siRNA towards either *Hif1a* or *Epas1*, both simultaneously, or a control siRNA, before activation of the HIF pathway with the hypoxia mimetic DP for 8 hours. Gene expression was analysed in response to the HIF knockdowns by qRT-PCR for *Rgs4*, characterised HIF targets *Vegf* [19] and *Dec1* [20], and the HIF-α encoding genes *Hif1a* and *Epas1*. The effect of siRNA knockdown is observed on targeted *Hif1a* and *Epas1* mRNAs, and confirms the specificity of the HIF-specific siRNAs. Interestingly, cells downregulated *Hif1a* while upregulating *Epas1* in response to DP treatment alone in control siRNA-transfected cells (Figure 3 and Figure S1). Significant negative effects on *Vegf* and *Dec1* response to DP were observed, although both of these genes were only sensitive to HIF-1α knockdown (Figure 3). However, *Rgs4* message levels were sensitive to knockdown of either HIF-α subunit, while knockdown of both *Hif1a* and *Epas1* produced a more dramatic inhibition of the response to DP similar to that observed with *Vegf* and *Dec1*. Similar results were achieved using alternative siRNA constructs designed to target HIF-1α and HIF-2α, although knockdown of *Hif1a* and *Epas1* was less efficient (Figure S1). Together these data are consistent with a contribution of both HIF-1α and HIF-2α to the induction of *Rgs4*.

### *Rgs4* Employs a Direct Transcriptional Response to Hypoxia

In order to determine whether *Rgs4* mRNA regulation by hypoxia is transcription-dependent, SK-N-BE(2)C cells were plated and treated with actinomycin D to block transcription, then subjected to a time-course of hypoxic or normoxic treatment before *Rgs4* transcript quantification by qRT-PCR. *Rgs4* mRNA degradation was not significantly different between normoxic and hypoxic samples when quantified relative to *Polr2a* (Figure 4A). *Rgs4* and *Polr2a* mRNA one-phase decay curves relative to total RNA were plotted, and *Rgs4* mRNA half-life was calculated to be between 3.5–4.0 hours in normoxia, and 4.0–4.6 hours in hypoxia within a 99% confidence interval. (Figure 4B,C). This small difference is similar to that observed with *Polr2a*, and cannot account for the larger reproducible hypoxic induction of *Rgs4* observed in Figure 2. Similarly, despite the presence of several conserved 3′UTR elements, the human *Rgs4* 3′UTR did not confer hypoxic response to a luciferase reporter gene in SK-N-BE(2)C cells (Figure 4D), further supporting a transcriptional induction of *Rgs4* by HIF in hypoxia.

To investigate whether the kinetics of *Rgs4* induction are consistent with other direct HIF targets, a hypoxia time-course was performed from 0 to 16 hours on the SK-N-BE(2)C cell line, and *Rgs4*, *Vegf*, *Dec1*, *Hif1a* and *Epas1* message levels were quantified using qRT-PCR. In addition, we tested for levels of *Mlh1* transcript, which is negatively regulated by the HIF-inducible gene product of *Dec1* [60], and therefore responds indirectly to the HIF pathway. The results demonstrate that *Rgs4* is significantly upregulated after 1 hour of hypoxic treatment, similar to the other direct HIF target genes *Vegf* and *Dec1* (Figure 4E), and is therefore likely to be a direct target of the HIF transcription factor. In contrast, the secondary HIF responder *Mlh1* displays delayed regulation, and is not significantly affected by hypoxia until 4 hours of treatment. Curiously, both *Hif1a* and *Epas1* mRNA levels were again regulated by DP treatment, in a similar timeframe to the *Mlh1* response, indicating that the HIF-encoding genes may be under indirect self-regulation in this cell-line.

### *Rgs4*-Proximal Genomic Sequences are Unresponsive to Hypoxia in Reporter Assays

Given the evidence for *Rgs4* being a direct transcriptional target of the HIFs, the *Rgs4* promoter was analysed for functional HREs that mediate the HIF-dependent hypoxic induction. We used the UCSC Genome Browser [58] to analyse the 32.8 kb sequence of the human genome containing the *Rgs4* promoter, coding sequence, introns and flanking regions. A search was conducted using the MBCS program [61] for the minimal HIF binding consensus sequence RCGTG [1], in both forward and reverse orientations, resulting in 27 hits, 4 of which result from 2 palindromic CACGTG sequences (Figure 5A). Conservation was assessed by comparison to mouse and rat genomic sequences using UCSC Genome Browser, at both primary sequence level and using the comparative genomics track of the Genome Browser. No putative HREs were identified which exhibited strong conservation across species, but we reasoned that enhancer sequences may have moved during evolution, as has been commonly reported for other enhancers [62], [63]. Alternatively, the *Rgs4* HRE may not conform to the consensus sequence. Therefore, to test the *Rgs4* locus for hypoxia responsive enhancers with an unbiased approach, the genomic regions analysed were divided into overlapping fragments and each amplified by genomic PCR (Figure 5A, primer sequences listed in Table S1), before subcloning into the pGL3 reporter plasmid. Genomic sequences including the *Rgs4* transcription start site replaced the existing promoter of pGL3, while all other sequences retained this minimal SV40 promoter to allow expression of Firefly Luciferase. One short region between sequences C and D could not be cloned either in isolation or in combination with adjacent regions C and D, and therefore could not be tested. However HRE-like sequences were not detected by bioinformatic analysis in this short region.

The 14 constructs corresponding to 32 kb were transfected into SK-N-BE(2)C cells together with a constitutive Renilla Luciferase expressing construct, and treated for 16 hours with hypoxia or normoxia before analysis by the Dual Luciferase System (Promega). No significant change in relative luciferase expression upon hypoxic treatment could be detected in any sequences tested (Figure 5B–E), similar to the empty pGL3-promoter control. In contrast, the positive controls demonstrated robust HRE-dependent activation. The hypoxic response of *Rgs4* mRNA is also observed in rat PC-12 cells, so the 5 kb promoter of the rat *Rgs4* gene was tested for hypoxic response in PC-12 cells, and similar results were obtained with no detectable hypoxic induction (data not shown). Thus, while *Rgs4* mRNA is clearly induced by hypoxia, this induction does not appear to be mediated by the 32 kb sequence surrounding the *Rgs4* gene, and the putative HREs contained within this sequence are not functional in this context.

## Discussion

The experiments presented here show that *Rgs4* is positively upregulated by hypoxia and hypoxia-mimetic chemicals DMOG and DP in the PC-12, Neuro-2A, SK-N-SH and SK-N-BE(2)C cell lines, and this regulation is sensitive to knockdown of HIF-1α and HIF-2α. In the human neuroblastoma cell lines, *Rgs4* is responsive to hypoxia in a similar timeframe to other direct targets of HIF transactivation, and a dissimilar timeframe to indirect HIF-responsive genes. Furthermore, *Rgs4* mRNA is not regulated by hypoxia post-translationally, and the highly conserved regions of the *Rgs4* 3′UTR do not confer hypoxia sensitivity to a reporter gene. These results are all consistent with HIF acting directly as a transactivator on *Rgs4* transcription.

Notably, these responses of *Rgs4* have only been seen in sympathetic cancer cell types such as neuroblastoma and pheochromocytoma, while previously characterised HIF target genes are regulated by hypoxia in a wider array of cell types, such as HUVECs. Contrary to other previous findings [59], we could not detect a hypoxic response for either *Rgs4* or *Rgs5* mRNA levels in HUVEC cells. This may be explained by our use of the more sensitive qRT-PCR expression quantification technique [59]. Our experiments demonstrate that *Rgs4* expression is regulated by hypoxia in only a subset of cell types that express *Rgs4*, and that hypoxic response is not common for all RGS encoding genes, but is specific for *Rgs4*.

*Rgs4* is expressed strongly in neural and vascular cell types, yet there are few well-elucidated physiological roles for its gene product. RGS4 protein can regulate signalling from the µ-opioid (morphine) and/or δ-opioid receptors depending on context [64]–[66], and also the D_2_R dopamine receptor [32], [33]. *Rgs4* dysfunction is thought to be related to disease states such as schizophrenia [30] and heart hypertrophy [29], [67], [68]. Increased *Rgs4* expression has also been noted during both epithelial and endothelial tubulogenesis [69]. Our experiments suggest that any role for hypoxia in regulating *Rgs4* function would most likely affect the neural roles of *Rgs4*, rather than its function in the vascular system, although a wider range of cell types needs to be tested. Importantly, hypoxia and ischaemia have also been associated with schizophrenia [34]–[37], and as such HIF regulation of *Rgs4* transcription in specific neural cell types may provide a molecular mechanism linking disrupted oxygen supply to schizophrenia.

We demonstrate that hypoxic regulation of *Rgs4* requires transcription and occurs in the same timeframe as other direct HIF transactivation targets. This implies a direct function for the HIF transcription factor in activation of *Rgs4* gene transcription. Other known hypoxia and HIF responsive genes are regulated by direct interaction between HIF and DNA at the regulated gene, and genomic HIF binding sites are often found within 2k b of a responsive promoter [22], which can commonly be characterised by reporter assay. However, no region of the human *Rgs4-*proximal locus tested confers hypoxic response as an enhancer when cloned into a luciferase reporter assay, hence no functional HRE could be detected in the 32 kb genomic sequence surrounding the transcription start site of the *Rgs4* gene. As there is some suggestion that *Rgs4* is also regulated by other bHLH/PAS family transcription factors such as NPAS4 [70], we searched published ChIP-Seq (Chromatin IP with deep Sequencing) data for NPAS4, CBP and PolII genomic binding sites at the mouse *Rgs4* locus [71]. Importantly, CBP is recruited by HIF-α subunits via their C-terminal transactivation domains in hypoxia, so DNA binding sites of NPAS4 and CBP may provide further insight into likely locations of hypoxia-responsive enhancers. Although this study used mouse cortical neurons undergoing depolarisation stimulation as a model, it is clear that mouse *Rgs4* is under the control of several distant enhancers, unlike other known target genes of NPAS4 such as *Drebrin*. Specifically, NPAS4 and p300 are associated with a number of sites distal to the *Rgs4* gene, with some sites of high occupancy for CBP and/or NPAS4 between 20 and 50 kb upstream of the *Rgs4* transcription start site, well beyond the region analysed in reporter assays. Therefore, it is likely that *Rgs4* may similarly be under the regulation of HIF acting from a distant *cis* binding site, probably interacting with the *Rgs4* promoter through DNA-looping. These sites may be shared with NPAS4, as DNA binding specificity is similar between HIF and NPAS4 [14], or they may be distinct. This model may provide an explanation for the differential regulation of *Rgs4* by hypoxia between neural and vascular cell types, as basal activation of the *Rgs4* promoter may be under different regulation to activation of distant enhancers. As such, a complex enhancer-promoter interaction may require additional cell type specific cofactors or chromatin organisation to enable transactivation of *Rgs4* transcription by HIF.

Whilst we demonstrate here that *Rgs4* is induced by hypoxia via a HIF-mediated mechanism in specific cell types, it is likely that a detailed ChIP-Seq approach for HIF-1 and HIF-2 using neuroblastoma cells will be required to definitively identify the DNA binding site for HIF. Such an approach can also identify other required factors or chromatin context, and thus may provide a mechanism for the cell-type specific regulation of *Rgs4*. It is also of interest to note that RGS4 protein stability has also been reported to be regulated by an alternative hypoxia-responsive pathway, the N-end rule pathway [38], supporting a complex regulation of *Rgs4* at multiple levels in response to hypoxia. Given that expression of *Rgs4* has been linked to diseases such as schizophrenia, where hypoxia is also thought to be a contributing factor, the hypoxic regulation of *Rgs4* in neural-like cells demonstrated here is of considerable relevance and supports further characterisation of the contributions of both *Rgs4* and HIF to this and other diseases.

## Supporting Information

Figure S1 **Knockdown of HIF-α subunits impairs response of** ***Rgs4*** **to hypoxia.** SK-N-BE(2)C cells were transfected twice with 50 nM siRNA (secondary sequences), before 8 hours 0.1% DMSO vehicle (V) or 100 µM DP treatment. Cells were then analysed for *Rgs4*, *Vegf*, *Dec1*, *Hif1a* and *Epas1* expression levels relative to *Polr2a* by qRT-PCR. Level of each target mRNA is shown, normalised to the DP-treated level of expression of each gene after negative control siRNA transfection. A representative of three independent experiments is shown.
(EPS)

Table S1 Sequences of primers used to clone regions of the human *Rgs4* gene and locus. PCR from genomic template was performed using listed primers, incorporating restriction enzyme sites. Products were then cloned to the enhancer, promoter or 3′ UTR site of pGL3 for luciferase reporter assay analysis (Figures 4 and 5).
(XLS)
